# Short-Term Exposure of Multipotent Stromal Cells to Low Oxygen Increases Their Expression of CX3CR1 and CXCR4 and Their Engraftment In Vivo

**DOI:** 10.1371/journal.pone.0000416

**Published:** 2007-05-02

**Authors:** Shih-Chieh Hung, Radhika R. Pochampally, Shu-Ching Hsu, Cecelia Sanchez, Sy-Chi Chen, Jeffrey Spees, Darwin J. Prockop

**Affiliations:** 1 Center for Gene Therapy, Tulane University Health Science Center, New Orleans, Louisiana, United States of America; 2 Stem Cell Laboratory, Department of Medical Research and Education, Veterans General Hospital-Taipei, Taipei, Taiwan; 3 Institute of Clinical Medicine, National Yang-Ming University, Taipei, Taiwan; New York University School of Medicine, United States of America

## Abstract

The ability of stem/progenitor cells to migrate and engraft into host tissues is key to their potential use in gene and cell therapy. Among the cells of interest are the adherent cells from bone marrow, referred to as mesenchymal stem cells or multipotent stromal cells (MSC). Since the bone marrow environment is hypoxic, with oxygen tensions ranging from 1% to 7%, we decided to test whether hypoxia can upregulate chemokine receptors and enhance the ability of human MSCs to engraft in vivo. Short-term exposure of MSCs to 1% oxygen increased expression of the chemokine receptors CX3CR1and CXCR4, both as mRNA and as protein. After 1-day exposure to low oxygen, MSCs increased *in vitro* migration in response to the fractalkine and SDF-1α in a dose dependent manner. Blocking antibodies for the chemokine receptors significantly decreased the migration. Xenotypic grafting into early chick embryos demonstrated cells from hypoxic cultures engrafted more efficiently than cells from normoxic cultures and generated a variety of cell types in host tissues. The results suggest that short-term culture of MSCs under hypoxic conditions may provide a general method of enhancing their engraftment in vivo into a variety of tissues.

## Introduction

Bone marrow contains several subpopulations of stem/progenitor cells that are capable of differentiating into various non-hematopoietic cells [1]–[4]. Among the best studied subpopulations are the cells that are isolated by their adherence to tissue culture surfaces and are referred to as mesenchymal stem cells or multipotent stromal cells (MSCs) [1], [2], [4]. MSCs have emerged as a promising tool for clinical applications such as tissue engineering and cell-based therapy, because they are readily isolated from a patient, can be expanded in culture, and have a limited tendency to form tumors. In addition, the cells tend to home to sites of tissue growth and repair, and to enhance tissue regeneration. Homing and engraftment of the cells is readily detected in rapidly growing embryos, including mouse [5], chick [6] and sheep [7], and following tissue injury, such as ischemic damage to heart [8], [9] and brain [10]. However, various studies have shown the degree of engraftment of MSCs in naive adult animals is very low [11].

Several attempts are currently being made to enhance the engraftment of stem/progenitor cells in vivo. Exogenously delivered or endogenously produced stromal cell-derived factor-1α (SDF-1α) plays a crucial role in recruitment of endothelial progenitor cells, bone marrow-derived stem cells, or embryonic stem cells to the ischemic tissues such as heart and brain [8], [12]–[14]. Engraftment of hematopoietic stem cells (HSCs) was also recently improved by either over-expression of the chemokine receptor CXCR4 or by an inhibitor for CD26, a protease that cleaves the NH_2_-terminus of CXCL12 (SDF-1α), a ligand for CXCR4 [15], [16]. Since bone marrow is hypoxic, we tested the possibility that short-term exposure of human MSCs to hypoxic conditions may increase their engraftment in vivo.

## Results

### Effects of hypoxia on apoptosis and subsequent expansion of MSCs

**Figure 1:**
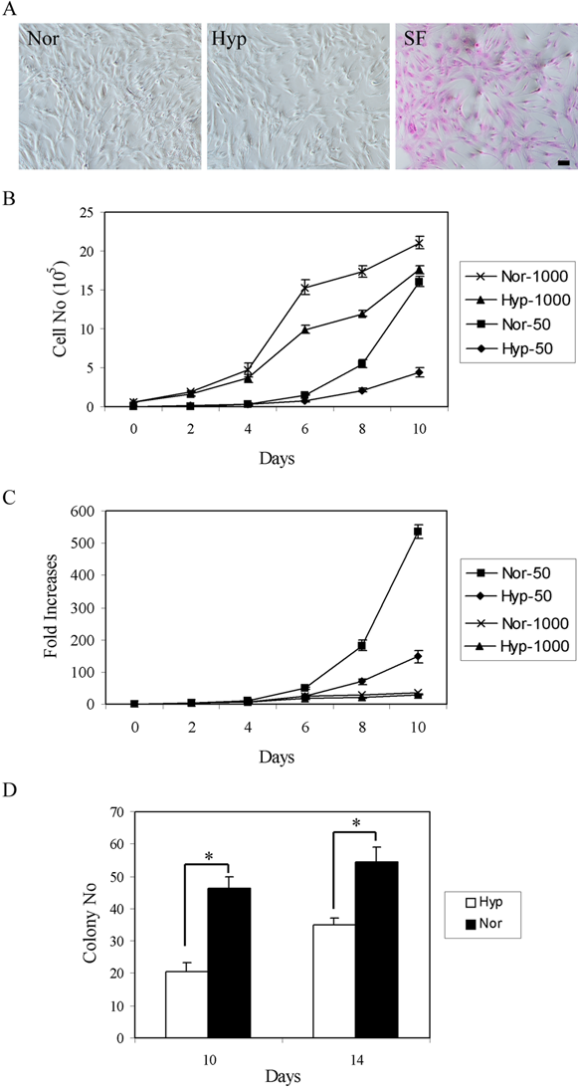
Effect of oxygen tensions and initial plating densities on apoptosis and expansion of MSCs. (A) The MSCs at passage 3 were subjected to normoxic (Nor), hypoxic (Hyp) or serum free conditions (SF) for 2 days. Apoptosis was evaluated by the APOPercentage assay and the apoptotic cells are stained as pink. (B,C) Passage 3 MSCs were plated on 60-cm2 dishes at 50 and 1,000 cells/cm2 and cultured in normoxic and hypoxic conditions. The cells were harvested and counted up to 10 days. (B) Total cell numbers per 60-cm2 dish and (C) fold increase are shown. Data are expressed as mean±standard deviation (n = 3). (D) Passage 3 MSCs were plated at 100 cells/60-cm2 dish, cultured in normoxic or hypoxic conditions for 10 or 14 days, and stained with Crystal Violet to give total colony count. Colony numbers, which indicate colony-forming efficiency (%), are shown. Data are expressed as mean±standard deviation (n = 3). (*, p<0.05, Student's t test).

We first determined whether exposure of MSCs to hypoxia increased apoptosis or limited their proliferative capacity in normoxic conditions. Assay of cultures with a dye that detects membrane alterations (phosphatidylserine flip) [17] did not reveal an increase in apoptosis after exposure of MSCs in CCM to 1% oxygen for 2 days (Figure 1A). In contrast, apoptosis was readily detected in control cultures that were incubated in serum-free medium for 2 days. With cells plated at 50 cells/cm^2^, MSCs grown under hypoxic conditions expanded 148-fold in 10 days, whereas control cells grown under normoxic conditions expanded 535-fold (Figure 1B,C). With cells plated at 1,000 cells/cm^2^, hypoxic MSCs expanded 29-fold in 10 days, whereas control cells expanded 35-fold (p<0.01; n = 3). In addition, after plating at 1.5 cells/cm^2^ to assay colony forming units, the hypoxic MSCs formed decreased numbers of single-cell derived colonies (p<0.01; n = 3) as compared to controls (Figure 1D). Therefore exposure to hypoxia decreased both the rate of proliferation and the colony forming capacity of the MSCs.

### Exposure to hypoxia decreases the capacity of MSCs to differentiate

**Figure 2:**
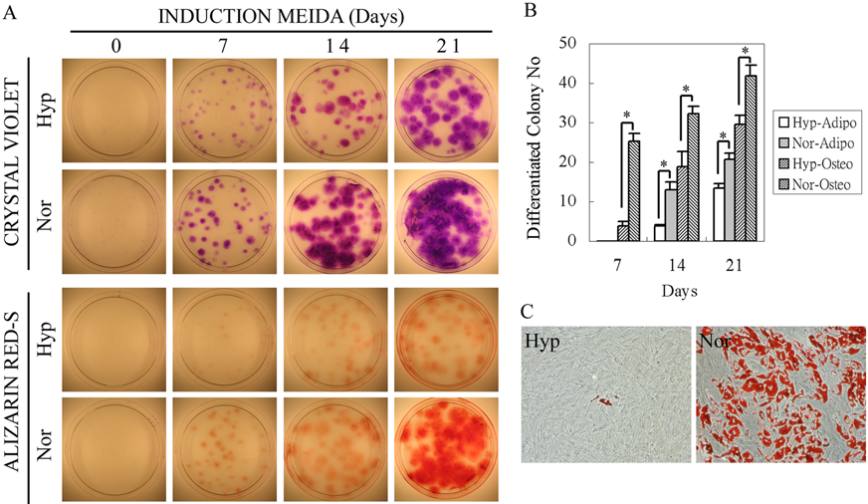
Hypoxia inhibits osteogenesis and adipogenesis. The MSCs were plated at 100 cells/60-cm2 dish, cultured in normoxic (Nor) or hypoxic (Hyp) conditions for 7 days, and replaced with osteogenic or adipogenic induction media for additional 7 to 21 days. (A) Total colonies stained with Crystal Violet (upper two panels) and osteogenic colonies stained with Alizarin Red-S (lower two panels). (B) The panel shows the numbers of Alizarin Red-S and Oil Red-O-positive colonies after culture in osteogenic and adipogenic induction media, respectively. Data are expressed as mean±standard deviation (n = 3). (*, p<0.05, Student's t test). (C) Cells were plated at 10,000 cells/cm2, and then cultured in adipogenic induction media under normoxic or hypoxic conditions. Oil Red-O staining was performed at 2 weeks after induction to visualize the level of fat production in the cells (red stain).

To examine the effects of hypoxia on differentiation, MSCs were plated at 1.5 cells/cm^2^ in a 60 cm^2^ dish, cultured in CCM under normoxic or hypoxic conditions for 7 days so that they generated small single-cell derived colonies. The cells were then continuously cultured under normoxic or hypoxic conditions and induced in osteogenic or adipogenic media for additional 7 to 21 days. Hypoxia decreased both the osteogenic differentiation and adipogenic differentiation as shown by the decrease in the numbers of the Alizarin Red-S and Oil Red-O positive colonies (Figure 2). The inhibition of osteogenic differentiation and adipogenic differentiation by hypoxia was also observed when MSCs were plated at a high density of 10^4^/cm^2^and induced to undergo adipogenic differentiation (Figure 2C) or osteogenic differentiation (not shown).

### Reoxygenation reverses the effects of hypoxia on proliferation and differentiation

To examine the effects of reoxygenation, cells were first plated at 50 cells/cm^2^ and incubated under either normoxic or hypoxic conditions for 8 days. The cells were then replated at varying densities and assayed for rates of proliferation and differentiation under normoxyic conditions. Over a 3 to 10 day period, the hypoxia-exposed cells proliferated at the same rate as controls (Figure S1A–C). Cells transferred to osteogenic medium after incubation in normoxic conditions, differentiated into osteoblasts at about the same rate as controls both in high density and low density cultures. Also cells transferred to adipogenic medium after incubation in normoxic conditions, differentiated into adipocytes at about the same rate as controls (Supplementary Figure 1D).

### Short-term exposure of MSCs to hypoxia increases expression of chemokine receptors

**Figure 3:**
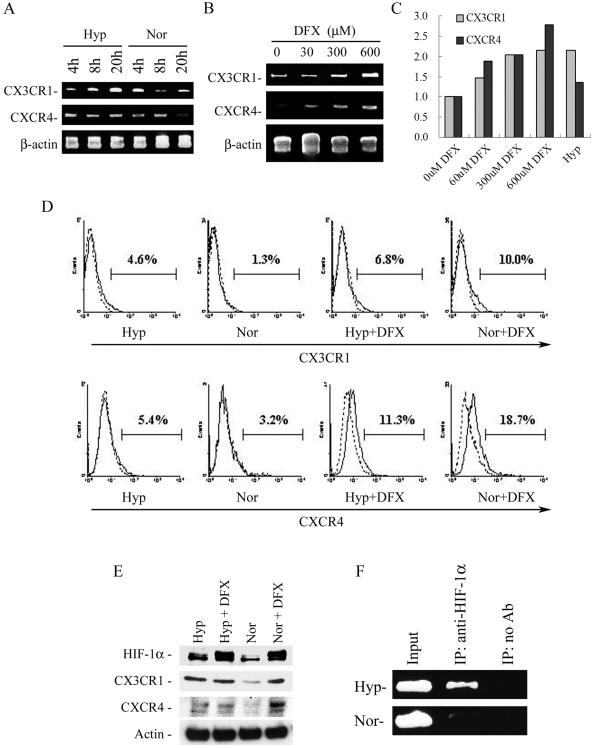
Effect of hypoxia on CX3CR1 and CXCR4 expression by early culture of MSCs. (A) The MSCs were cultured for 4–20 h in normoxic (Nor) or hypoxic (Hyp) conditions, as indicated. Total RNA was analyzed by RT-PCR for CX3CR1, CX3CR4, and b-actin mRNA expression. (B) Total RNA from MSCs cultured for 20 h in the presence of increasing concentrations of DFX was analyzed by RT-PCR for CX3CR1 and CXCR4 mRNA expression. (C) Quantitaive RT-PCR for CXCR4 and CX3CR1 mRNA expression at 20 h of treatment. The vertical axis represents the relative ratio of indicated condition to normoxic condition without the addition of DFX.. (D) Surface expression of CXCR4 and CX3CR1 were determined by flow cytometry using a mouse mAb anti-CXCR4 and a rat mAb anti-CX3CR1, respectively (continuous line). Irrelevant antibody is indicated by the dotted line. The results are representative of three independent experiments. (E) Hypoxia and DFX-treated MSCs were cultured for 24 h in the indicated conditions and analyzed by western blot for HIF-1α, CXCR4, CX3CR1, and actin protein levels. (F) HIF-1α binding to the CX3CR1 promoter. The MSCs were cultured for 4 h in normoxic or hypoxic condition. ChIP was performed with or without rabbit antibody specific for HIF-1α. There is no difference between PCR of the CX3CR1 promoter using input chromatin for normoxic and hypoxic cells. However, a band was noted for hypoxic cells but not for normoxic cells, when PCR of the CX3CR1 promoter was performed using immunoprecipitation with HIF-1α antibody.

A recent report demonstrated that chemokine receptors expressed on MSCs mediated their migration to tissues [18]. Therefore, we harvested passage 2 or 3 MSCs directly from the frozen vials and plated them at 1,000cells/cm^2^, incubated them under hypoxic or normoxic conditions for 4 to 20 hr, and assayed extracted mRNAs by RT-PCR for CX3CR1 and CXCR4, receptors for fractalkine and SDF-1α. Under normoxic conditions, the cells continued to express CX3CR1 but the levels of CXCR4 decreased by 20 h (Figure 3A). Under hypoxic conditions, the expression of CX3R1 increased with time. Expression of CXCR4 was unchanged (Figure 3A). However, the levels of both CX3CR1 and CXCR4 increased in a dose-dependent manner in the presence of the iron chelator desferroxamine (DFX), which directly inhibits prolyl hydroxylases with concomitant HIF-1α stabilization (Figure 3B). These data were reproduced by Quantitative RT-PCR (Figure 3C) and further RT-PCR assays indicated that both normoxic and hypoxic cells expressed about the same levels of other CC, CXC, CX3C, and CX chemokine receptors (not shown).

Increased expression of CX3CR1 and CXCR4 by hypoxic MSCs was also confirmed at the protein level by flow cytometry (Figure 3D) and western blots (Figure 3E). As shown in Figure 3C and 3D, after culture in hypoxic conditions for 20–24 h, MSCs contained higher levels of CXCR4 and CX3CR1 than control cells, and DFX treatment, either in hypoxic or normoxic conditions, achieved much higher levels. For reasons that were not apparent, DFX had a slightly greater effect on cells incubated under normoxic than hypoxic conditions (Figure 3D and 3E). We also detected an increase in protein level of hypoxia-inducible factor-1α (HIF-1α) in hypoxia or DFX-treated MSCs (Figure 3E); however no difference could be detected in mRNA levels (not shown). These data support previous reports that HIF-1α is regulated at the protein level, not at the mRNA level [19].

Since induction of CXCR4 by hypoxia has previously been shown to be driven by HIF-1α [19], [20], we explored whether the HIF-1α also drives expression of CX3CR1. To obtain direct evidence for the interaction between HIF-1α and the CX3CR1 promoter, we used the ChIP assay to measure the HIF-1α recruitment to the CX3CR1 promoter. Although no interaction between HIF-1α and the CX3CR1 promoter was observed in normoxic condition, recruitment of HIF-1α to the CX3CR1 promoter was clearly detected at 4 h after cultured in hypoxic condition (Figure 3F). This result is consistent with the induction of CX3CR1 expression in hypoxic condition and in DFX-treated normoxic condition (Figure 3A–D). Overall, these data demonstrate the involvement of HIF-1α in the induction of the CX3CR1 promoter.

### Increased migration of MSCs in response to chemokines after short-term exposure to hypoxia

**Figure 4:**
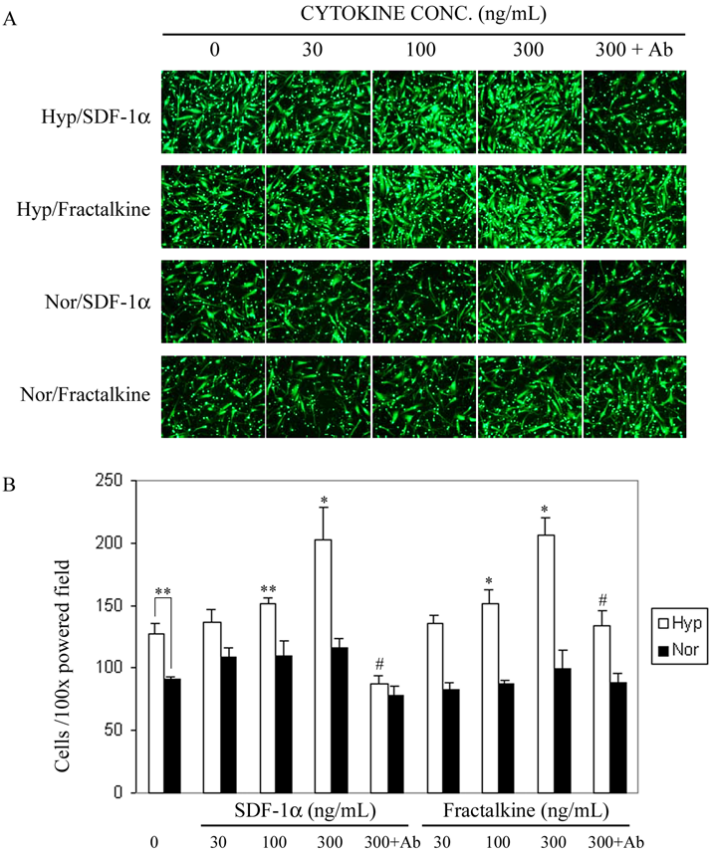
Effect of hypoxia on the chemotactic response of MSCs to SDF-1a and fractalkine. (A) Cells were cultured for 22 h in normoxic (Nor) or hypoxic (Hyp) conditions. Migration of MSCs was assayed by chemotaxis microchamber technique. Effect of hypoxia on the chemotactic response of MSCs to increasing concentrations of SDF-1a (0–300 ng/mL) and fractalkine (0–300 ng/mL) was assessed by counting the total number of cells in 5 power fields. For inhibition studies of MSCs migration, MSCs were preincubated with 10 mg/mL of anti-CXCR4 or fractalkine-containing medium was preincubated with 10 mg/mL of anti-fractalkine. (B) Results are mean±SD of five power fields. (*, p<0.01, **, p<0.05 as compared with control without cytokine, #, p<0.01 as compared with that with cytokine added at 300 ng/mL, Student's t test).

To investigate the effect of hypoxia on cell migration, we used a modified Boyden chamber method to examine the migration of MSCs over a 14 h period, after the cells were cultured under hypoxic conditions (Figure 4A). Exposure of MSCs to hypoxic conditions for 1 day significantly increased their migration in the absence of chemokines (Figure 4B). The hypoxic cells also migrated more rapidly in response to the chemokines SDF-1α (CXCL12, ligand of CXCR4) and fractalkine (CX3CL1, ligand of CX3CR1) in a dose dependent manner. The blocking antibodies anti-CXCR4 or anti-CX3CL1 significantly decreased the chemotaxic effects of chemokines (Figure 4B).

### Increased engraftment of MSCs into chick embryo after short-term exposure to hypoxia

**Figure 5:**
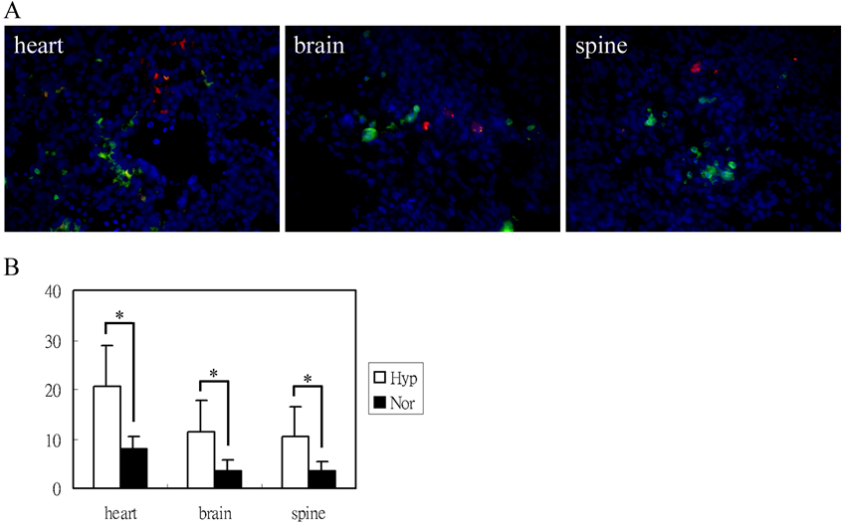
Dye-labeled MSCs transplanted in 2-day-old chicken embryo and detected in organs of 5-day-old chicken embryo by epifluorescence microscopy. (A) Examples of hypoxia-exposed/CMFDA-labeled (green) and normoxic condition-exposed/CMTMR cells (red) in the heart, brain or spine area. 4',6-Diamidino-2-phenylindole (DAPI) stain was used to identify nuclei (mag: 400×). (B) Cells per 400×powered field. Results are mean±SD of more than 20 power fields from different embryos. (*, p<0.05, Student's t test).

To test the ability of MSCs to engraft in vivo, MSCs or PBS were infused into the day-2 chick embryos. Evaluation for engraftment was first carried out by real-time PCR assays to detect a human-specific fragment of the α-satellite DNA on human chromosome 17 in chick embryos [21]. Seventeen of 28 embryos tested were positive for human chromosome 17. The degree of chimerism with hypoxia-exposed MSCs was slightly higher (mean of 89/10,000 cells with range of 1.5 to 600) than with normoxic MSCs (mean of 30/10,000 cells with range of 0.9 to 170) but the results were not statistically significant, because of the highly variable success rate of the microsurgery in the embryos. To overcome the variability, we used a competitive engraftment assay in which hypoxic MSCs and normoxic MSCs were labeled with CMFDA and CMTMR, respectively. To test the suitability of this assay for the purpose, A 1∶1 mixture of CMFDA-labeled and CMTMR-labeled MSCs were incubated under normal expansion condition for 3 days. The labeling of cells with both vital dyes did not block cell proliferation and the dyes were brightly observed after the fixation procedures (Figure S2). After mixtures of equal numbers of both cells were infused into the embryos, greater numbers of the CMFDA-labeled hypoxic cells were found in the tissues of the day-5 chick embryos, which included heart, brain, liver and spinal cord (Figure 5A, B).

### Differentiation of MSCs into heart and brain tissues of chick embryos without cell fusion

**Figure 6:**
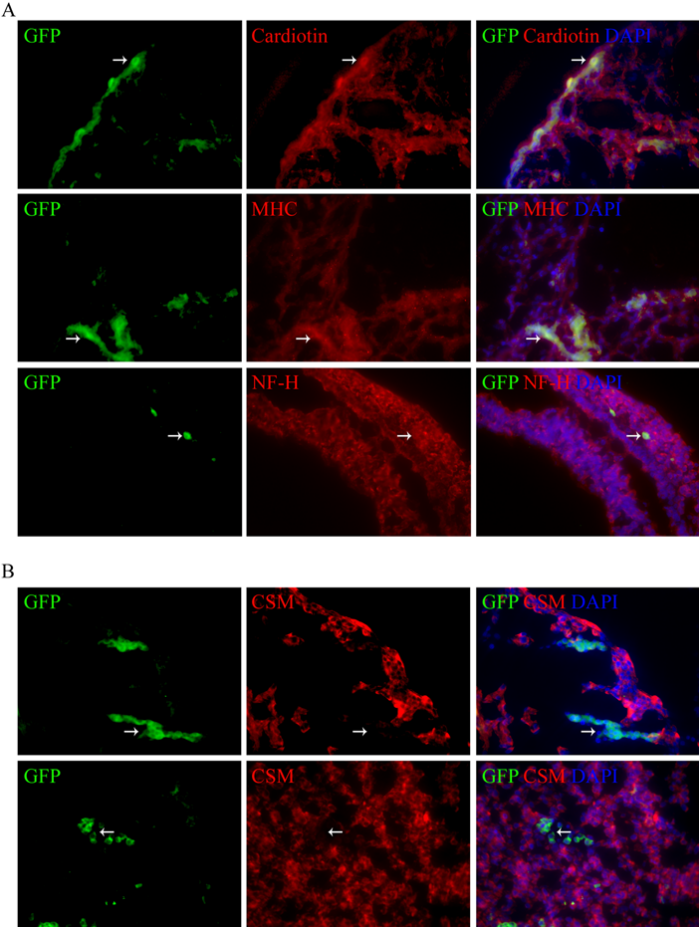
Epifluorescent immunohistology of sections from chick embryos infused with GFP+MSCs. (A) Cells (arrows) positive for GFP and cardiotin in heart (upper row); cells (arrows) positive for GFP and a-myosin heavy chain (MHC) in heart (middle row); cells (arrows) positive for GFP and NF-H in brain (bottom row). (B) Cells (arrows) positive for GFP, but negative for a chicken-specific marker (CSM) in heart (upper row) or brain area (lower row). DAPI stain was used to identify nuclei (mag: 400×).

For examination of differentiation potential of hypoxia-exposed hMSC, GFP+MSCs transduced with a lentiviral vector expressing GFP [22] and enriched by FACS (more than 95% positive for GFP) were infused into the day-2 chick embryos. In histological sections of the embryos harvested 3 days later, GFP+cells were detected in multiple developing organs of the embryos, including heart, liver, brain, and spinal cord. The most common site of appearance of the GFP+cells was the heart and spinal cord, apparently because the cells were infused into somites that overlay the dorsal aorta. Immunostaining of sections of heart demonstrated that some of the GFP+cells expressed cardiotin, a protein found in the longitudinal sarcoplasmic reticulum of mature cardiomyocytes (Figure 6A). In addition, some stained for the cardiac-specific protein α-myosin heavy chain (Figure 6A). Some GFP+cells in the subventricular zone of the brain expressed the neuronal marker neurofilament H (NF-H) (Figure 6A). Apparently because of the early stage of development, no astrocyte-lineage differentiation was found by staining with GFAP. To assess whether cell fusion could contribute the lineage differentiation of GFP+MSCs, we compared the expression of GFP with that of the chicken specific marker, 8F3, using immunofluorescence (Figure 6B). We observed a complete segregation of human and chicken cells at 3 days after implantation; no cell expressed both markers. We also searched for the presence of double nuclei using fluorescent nuclear stains under microscopy. Of 3,120 human cells in 12 embryos, none contained more than one nucleus. In most of the human cells, the human nucleus was readily identified morphologically because of its larger size (Figure 6B). These data were consistent with our previous observations that adult stem cells from rat marrow engrafted and partially differentiated into heart and brain tissues without evidence of cell fusion.[6]

## Discussion

In this report, we demonstrated that MSCs from human bone marrow can be expanded in vitro under hypoxic conditions. We used different plating densities and assays on single-cell derived colonies to evaluate the effects of hypoxia on proliferation and differentiation capacity of MSCs. We found MSCs incubated under hypoxia had decreased rates of proliferation and decreased capacities for both osteogenic and adipogenic differentiation. However, there was no increase in apoptosis under the conditions employed. The results were consistent with some previous reports [23], but not with other reports [24], [25]. The discrepancies may be in part be explained by the variation in the oxygen tension and the duration of hypoxic culture that has been used in each study. The other reason to explain the discrepancies is the variation of the system that has been used in each study to control the oxygen level. To minimize the fluctuations in oxygen levels during long-term incubation, we used an incubator that is equipped with two gas sensors, one for CO_2_ and the other for O_2_, and the O_2_ concentration was controlled using delivery of N_2_ generated from a liquid N_2_ tank.

Recent reports established that the chemokine receptors CXCR4 and CX3CR1 are important for mediating specific migration of MSCs to bone marrow or damaged tissues [26], [27]. Also, previous reports established that CXCR4 was up-regulated by hypoxia in monocytes, endothelial cells, and cancer cells [20] by the stabilization and activation of HIF-1α protein [19]. We found that both CXCR4 and CX3R1 are upregulated by exposure of MSCs to hypoxia or a reagent that mimics the response to hypoxia. We also demonstrated, for the first time, that the upregulation of CX3R1 is also dependent on HIF-1α. The upregulation of CXCR4 and CX3CR1 probably explained the enhanced migration of hypoxia-exposed MSCs in a modified Boyden chamber in response to SDF-1α and fractalkine, and their enhanced in vivo engraftment.

Xenotypic grafting into chick embryo has been a powerful tool in studies of engraftment and *in vivo* differentiation potential of progenitor or stem cells. Recent reports have shown that human embryonic stem cells, rat mesenchymal stem cells, mouse neural stem cells, and human haematopoietic stem cells can integrate into the chick embryo and differentiate into various cell types with no apparent fusion to the host chicken cells [6], [28]–[30] . The data presented here and previously [6] demonstrated that after MSCs were injected into early stage chick embryos, about two-thirds of the surviving embryos harvested 3 days later contained human cells in multiple tissues. The average percentage of chimerism and the number of dye-labeled human cells were about three-fold greater with hypoxia-exposed than with normoxic MSCs. Therefore, short-term exposure of MSCs to the hypoxic conditions normally found in marrow may prove a simple means of enhancing engraftment of the cells in vivo.

## Materials and Methods

### Reagents

Human recombinant SDF-1α and fractalkine were from PeproTech (Rocky Hill, NJ; http://www.peprotech.com). Desferrioxamine (DFX) was purchased from Sigma-Aldrich (St. Louis, MO; http://www.sigmaaldrich.com). The monoclonal antibodies against hypoxia inducible factor-1α, (HIF-1α, Cat # 241809, 1∶500 dilution), CXCR4 (Cat # 12G5, 1∶500 dilution) and its isotype control antibody were purchased from R&D systems (Minneapolis, MN; http://www.rndsystems.com). The monoclonal antibody (mAb) against actin was purchased from Chemicon (MAB1501, Temecula, CA; http://www.chemicon.com). The rabbit polyclonal antibody against CX3CR1 and its isotype control antibody were purchased from eBioscience (San Diego, CA; http://www.ebioscience.com). The antibodies were used for both FACS and Western Blot analyses.

### Culture of Human MSCs

Frozen vials of passage 2 to 4 of three extensively characterized human MSCs [31] were used at passage 2 to 4 were obtained from the Tulane Center for Preparation and Distribution of Adult Stem Cells (http://www.som.tulane.edu/gene_therapy/distribute.shtml).

The cells (about 1 million) were plated on a 15 cm diameter plate in complete culture medium (CCM) that consisted of alpha minimal essential medium (αMEM; GIBCO/BRL; Carlsbad, CA; http://www.invitrogen.com), 17% fetal bovine serum (FBS) lot selected for rapid growth of MSCs (Atlanta Biologicals, Inc.; Norcross, GA; http://atlantabio.com/default.htm), 100 units/ml penicillin, 100 µg/ml streptomycin, and 2 mM L-glutamine (GIBCO/BRL). After incubation for 24 hr, the viable adherent cells recovered with 0.25% trypsin and 1 mM EDTA at 37°C for about 5 min. The cells were replated at 100 cells/cm^2^, incubated in CCM with a change in medium every 2 to 3 days, and recovered as passage 2 cells after they reached about 80% confluency in 8 to 9 days. For hypoxic conditions, cells were cultured in a gas mixture composed of 94% N_2_, 5% CO_2_, and 1% O_2_ or treated in a medium containing 60–600 µM of DFX that mimics the hypoxic conditions by inhibiting the hydroxylation of a prolyl residue that is essential for the ubiquitination of HIF-1α [20]. For maintenance of the hypoxic gas mixture, we used an incubator with two air sensors, one for CO_2_ and the other for O_2_; the O_2_ concentration was achieved and maintained using delivery of nitrogen gas (N_2_) generated from a liquid nitrogen tank. If O_2_ percentage rose above the desired level, N_2_ gas was automatically injected into the system to displace the excess O_2_. For reoxygenation experiments, cells were first cultured at 50 cells/cm^2^ under normoxic or hypoxic conditions, recovered at day 8, and reseeded at 50 or 1,000 cells/cm^2^ and cultured under normoxic conditions. The growth curves and differentiation potentials of cells from normal culture conditions and hypoxic conditions were compared. The APOPercentage Apoptosis Assay (US Vendor: Accurate Chemical&Scientific Corporation., Westbury, NY; www.biocolor.co.uk) which detects membrane alterations (phosphatidylserine flip), was used to quantify apoptosis, according to the manufacturer's instructions.

### MSC Differentiation

For low density differentiation of colonies, MSCs (passage 3) were plated at 1.5 cells per cm^2^ in a 60-cm^2^ dish and cultured in CCM for 7 days. The medium was then replaced with osteogenic or adipogenic differentiation medium, and the cells were cultured for an additional 21 days. The osteogenic medium consisted of CCM supplemented with 10^−8^ M dexamethasone (Sigma), 50 µg/ml ascorbate-2 phosphate (Sigma), and 10 mM ß-glycerophosphate (Sigma). The adipogenic medium consisted of CCM supplemented with 0.5 µM dexamethasone (Sigma), 0.5 mM isobutylmethylxanthine (Sigma), and 50 µM indomethacin (Sigma). The osteogenic cultures were fixed in 10% formalin for 15 min and stained with 2% Alizarin Red-S for 30 min. Plates were washed 4 times with PBS and dried, and the numbers of Alizarin Red-S positive colonies were counted. The adipogenic cultures were fixed in 10% formalin for over 1 h and stained with fresh Oil Red-O solution for 2 h. Plates were washed three times with PBS and dried, and the numbers of Oil Red-O positive colonies were counted. Separated osteogenic and adipogenic cultures were also stained with crystal violet, and the number of total cell colonies were counted. For high density differentiation, human MSCs (passage 3) were seeded at 10,000/cm^2^ and differentiation was induced the next day by replacement of CCM with osteogenic or adipogenic medium. The cultures were washed with PBS and the differentiation induction medium was changed every three days. Staining was done as described for the low density cultures.

### RT-PCR

Total cellular RNA was extracted (RNAqueous Total RNA Isolation Kits; Ambion; Austin, TX; http://www.ambion.com), and cDNAs were generated by reverse transcription of 1–2 µg of cellular RNA (M-MLV Reverse Transcriptase; Invitrogen; Madison, WI; http://www.invitrogen.com), according to the manufacturer's instruction. Briefly, 1–2 µg of total RNA was reverse transcribed in a 20 µl reaction mixture. The same amount of template was subjected to 25 cycles of PCR amplification by using β-actin primers as an internal standard so that quantitation of mRNA levels could be corrected among the samples. PCR of CX3CR1 and CXCR4 was performed for 30 cycles. . Each cycle consisted of denaturing at 94°C for 30 seconds, annealing at 60–62°C for 30 seconds, and elongating at 72°C for 30 seconds, with an additional 7 minute incubation at 72°C after completion of the last cycle. PCR primers were designed from the published sequence of each cDNA as follows [26]: CX3CR1 (491 bp), sense: 5′-tccttctggtggtcatcg-3′ antisense: 5′-tgtgcattgggtccatca-3′; CXCR4 (260 bp), sense: 5′-agctgttggctgaaaaggtggtctatg-3′, antisense: 5′-gcgcttctggtggcccttggagtgtg-3′; β-actin (283 bp), sense: 5′-tcatgaagtgtgacgttgacatccgt-3′ antisense: 5′-cttagaagcatttgcggtgcacgatg-3′. The samples were then separated on a 1% agarose gel in the presence of ethidium bromide and PCR products detected and documented by using a GelDoc 1000 apparatus (Bio-Rad Laboratories, Hercules, CA). Quantitative RT-PCR was performed using the ABI assay (Applied Biosystems, Foster City, CA) on commercial primers, probes (CX3CR1: Hs00365842_m1; CXCR4: Hs00607978_s1) and Taqman universal PCR master mix on the ABI7700 realtime PCR machine according to the manufacturer's instructions.

### Flow Cytometry Analysis

Suspensions of MSCs lifted with EDTA alone were washed and incubated for 30 min at 4°C with monoclonal antibodies to human CXCR4, to human CX3CR1, or to control mouse IgG, followed by incubation for 30 min at 4°C with FITC-conjugated, isotype-matched affinity-purified, goat anti-mouse IgG. The sample was analyzed in Cytomics FC 500 (Beckman Coulter; Miami, FL; http://www.beckman.com).

### Western Blotting

Nuclear fraction was prepared for Western blotting of HIF-1α, and cytoplasmic fraction was prepared for Western blotting of CX3CR1 and CXCR4. Briefly, cells were prepared and lysed in buffer (M-PER for total cell lysate or NE-PER for nuclear fraction; Pierce Biotechnology, Rockford, IL; http://www.piercenet.com) supplemented with protease inhibitor cocktail (Halt; Pierce Biotechnology) and protein concentration was determined (Micro BCA Kit; Pierce Biotechnology). The cell lysate (20 µg of protein) was fractionated by sodium dodecyl sulfate–polyacrylamide gel electrophoresis (NuPAGE, 4–12% Bis-Tris gels; Invitrogen, Carlsbad, CA). The sample was transferred to a filter (Immobilon P; Millipore, Bedford, MA) by electro-blotting (XCell II Blot Module; Invitrogen). The filter was blocked for 1 h with TBS containing 5% nonfat dry milk and 0.05% Tween 20 and then incubated overnight at 4°C with the primary antibody. The filter was washed 3 times for 10 minutes each with TBS containing 0.1% Tween 20. Bound primary antibody was detected by incubating for 1 h with horseradish peroxidase-conjugated goat anti-mouse or anti-rabbit IgG (Pharmingen; San Diego, CA; http://www.pharmingen.com). The filter was washed and developed using a chemiluminescence assay (West-Femto Detection Kit; Pierce Biotechnology).

### Chromatin Immunoprecipitation (ChIP) Assay

To demonstrate the binding of HIF-1α protein to the promoter of CX3CR1, the ChIP assay was performed with a commercial kit (Upstate Biotechnology; Lake Placid, NY; www.upstatebiotech.com) using the manufacturer's protocol with minor adjustments. The MSCs were grown to confluence, incubated in air or 1% O_2_ for 4 h, and formaldehyde was added directly to culture medium to a final concentration of 1% followed by incubation for 20 min at 37°C. The cells were washed at 4°C in PBS, lysed on ice for 10 min in lysis buffer [10 mM Tris HCl, pH 8.0, 1% SDS] containing phosphatase and protease inhibitors. The lysates were sonicated three times for 30 s (Branson Sonifier 450), and the debris was removed by centrifugation. The supernatant was split into several aliquots. Sonication was optimized to produce average DNA fragments of 1 kb. One aliquot of the soluble chromatin was saved at −20°C for preparation of input DNA, and the remainder was diluted 10 times in immunoprecipitation (IP) buffer [10 mM Tris HCl, pH 8.0, 0.1% SDS, 1% Triton X-100, 1 mM EDTA, and 150 mM NaCl] containing phosphatase and protease inhibitors, and incubated overnight (4°C) with polyclonal antibody to human HIF-1α (Novus Biologicals; Littleton, CO; www.novus-biologicals.com). DNA–protein complexes were isolated on salmon sperm DNA linked to protein A agarose beads and eluted with 1% SDS, and 0.1 M NaHCO_3_. Cross-linking was reversed by incubation at 65°C for 5 h. Proteins were removed with proteinase K, and DNA extracted with phenol/chloroform, redissolved and PCR-amplified with CX3CR1 promoter primers, sense: 5′-attcagcagatatagggcag-3′; and antisense: 5′-acagtcagctctcattaatg-3′ (reverse), which gives a product length of 202 bp. The cycling parameters are 35 cycles, with each cycle consisting of denaturing at 94°C for 30 seconds, annealing at 60°C for 30 seconds, and elongating at 72°C for 30 seconds, with an additional 7-minute incubation at 72°C after completion of the last cycle.

### Chemotaxis Assay

Cells were labeled with a vital dye, either CMFDA (green, 492 ex, 516 em, Molecular Probes) or CMTMR (red, 540 ex, 566 em, Molecular Probes; Eugene, OR; http://www.probes.com) at a final concentration of 1–10 µM in pre-warmed Hank's Balanced Salt Solution (HBSS) for 10 min at 37°C. The medium was replaced with pre-warmed CCM and incubated for 30 min at 37°C. Cells were then washed three times with PBS, dislodged with 0.5 mM EDTA and dispersed into homogeneous single-cell suspensions in serum-free and phenol red-free αMEM to a concentration of 10^5^ cells/300 µl. To assess chemotaxis, a modification of the Boyden chamber method was used. CMFDA-labeled cells (10^5^) were added to each insert of 24-Multiwell plate (BD Falcon™ HTS FluroBlock™ Insert System, 8-µm pore size), and 1 ml of phenol red-free αMEM with increasing concentrations of SDF-1α (PeproTech) or fractalkine (PeproTech) was added to the lower compartments. For blocking the activity of SDF-1α, cells were incubated for 30 min on ice with antibody against human CXCR4 (12G5, 10 µg/mL, R&D) before loading on insert. For blocking the activity of fractalkine, antibody against CX3CL1 (81506, 10 µg/mL, R&D) was added into the medium for 30 min before loading in the lower compartments. Migration was allowed to proceed for 14 to 20 h at 37°C in air. Cells that had migrated to the lower surface of the filter were counted under a fluorescence-equipped microscope at 100×magnifications. The average number of migrating cells per field was assessed by counting at least four random fields per filter. Data points indicate the mean obtained from three separate chambers within one representative experiment.

### Infusion of MSCs in Chick Embryos

Chick embryos were removed from storage at 4°C and incubated at 38°C for 40–48 h until they developed to about Hamilton Hamburger stages 12–14. To lower the embryo, 2 ml of albumin was removed from the tapered end of the egg by using an 18-gauge needle and a syringe. An oval opening approximately 1 inch long was made in the shell on the broad side to view the embryo. Filtered 5% India Ink (Pelikan) in PBS was injected under the embryo to increase the contrast. The two or three most recently formed somites of 12–20 somites present in the chick embryos were crushed or removed by using a titanium needle (McCrone Microscopes, Westmount, IL). Before infusion into chick embryos, human MSCs were plated at 1,000 cells/cm^2^ and incubated for 1 day under hypoxic or normoxic conditions. A 1∶1 mixture of CMFDA-labeled (hypoxic cells) and CMTMR-labeled MSCs (normoxic cells) were suspended at 4,000 cells per µl in PBS at 4°C, and 10–15 µl of cell suspension was injected into the space created by removing the somites. An equal volume of PBS was injected into control embryos. For gene marking to trace the fate of transplanted cells, we also transduced MSCs with lentiviral vectors with the pWPT-GFP construct [22] donated by the Trono lab (Swiss Institute of Technology Lausanne, Lausanne, Switzerland). The embryos were harvested for immunofluorescence or real-time analysis at 3 days after the infusion, at which time they had developed to stage 25–26 and had begun to develop organs such as heart, brain and liver.

### Real-time PCR Analysis for the Human 17 Chromosome

Embryos were harvested, rinsed with PBS, fixed in 4% paraformaldehyde in PBS at 4°C overnight, transferred to 30% sucrose solution, followed by flash freezing, and cutting into 20 µm sections with a cryostat. Every 2nd and 3rd section was mounted on a slide for microscopy and the remaining sections were collected into a tube for real-time PCR analysis. For PCR assays, sections were incubated with 1 ml of proteinase K solution (0.4 mg/mL proteinase K/10 mg/mL SDS in Tris buffer at pH 7.4; Sigma) at 55°C for 24 h. The DNA was extracted by using phenol/chloroform (Sigma). The precipitated DNA was purified by using DNeasy tissue kit (Qiagen, Chatsworth, CA). Approximately 300 ng of chicken embryo DNA was used to analyze the engraftment of human MSCs by using a Universal PCR mix (Applied Biosystems, Foster City, CA) and primers and probes for a human-specific 850-bp fragment of the alpha-satellite DNA on human chromosome 17. The primers and probe set used were sense: 5′-caagtcaagcgccccatgaa-3′ and antisense: 5′-ttgagccaacttgtgcctctctc-3′ and probe: 5′-FAM-tgcatttatggtgtggtcccgcg-TAMRA-3′, respectively [21]. The conditions were initial denaturation at 94°C for 10 min, then 40 cycles with denaturation at 94°C for 1 min and annealing at 60°C for 1 min. Standards were run simultaneously by using pure uninjected chicken embryo DNA and purified DNA from MSCs.

### Microscopy

Sections from the embryos were analyzed both by epifluorescence of GFP and immunolabeling. Monoclonal antibody to cardiotin, α-heavy-chain myosin, NF-H, GFAP or MSOP (Chemicon Temecula, CA, and Abcam, San Antonio, TX) was incubated under conditions recommended by the supplier overnight at 4°C. For detecting fusion with host cells, slides were also incubated with a mAb against chicken specific antigen (8F3, Developmental Studies Hybridoma Bank at the University of Iowa). The slides were washed three times for 5 min with PBS at room temperature. They were incubated with Alexa-594-tagged (Molecular Probes) secondary anti-mouse antibody at 1∶1,000 dilution for 1 h. Controls included omitting the primary antibody. Slides were evaluated by using epifluorescence microscope (Eclipse 800; Nikon).

## Supporting Information

Figure S1 Reoxygenation reverses hypoxic effects on cell proliferation and differentiation. Passage 3 MSCs were plated at 50 cells/cm2, cultured in normoxic (Nor) or hypoxic (Hyp) conditions for 8 days, replated at (A) 50, (B) 1,000, (D-High Density panels) 10,000 cells/cm2 and (C, D-Low Density panels) 100 cells/60-cm2 dish and then cultured under normoxic conditions. (A, B) The cells were harvested and counted at 10 days (A) or at 3, 5 and 10 days (B). Graphs represent the fold increase in cell number per 60-cm2 dish. (C) Number of Crystal Violet-positive colonies formed at 10 and 14 days. Data are expressed as mean±standard deviation (n = 3). (*, p<0.05, Student's t test). (D-High Density panels) Cells were replated with induction medium the next day, cultured for additional 21 days and stained with Alizarin Red-S or Oil Red-O. (D-Low Density panels) Cells were cultured in CCM for 7 days and replated with adipogenic or osteogenic induction medium for additional 21 days. Osteogenic cells/colonies stained with Alizarin Red-S and adipogenic cells/colonies stained with Oil Red-O.
(9.99 MB TIF)

Figure S2 Detection of fluorescence after labeling with the vital dyes. Cells recovered from hypoxic and normoxic cultures were labeled with CMFDA and CMTMR, respectively. CMFDA-and CMTMR-labeled cells were then mixed at the ratio of 1 to 1 and incubated under a normal expansion condition. The cells were fixed and observed with an epifluorescence microscope 3 days later (200×magnification).
(4.31 MB TIF)
