# Supplementary figures and images for: Short-Term Exposure of Multipotent Stromal Cells to Low Oxygen Increases Their Expression of CX3CR1 and CXCR4 and Their Engraftment In Vivo
Source: PLoS One. 2007 May 2;2(5):e416. doi: 10.1371/journal.pone.0000416 (PMC1855077; doi:10.1371/journal.pone.0000416)

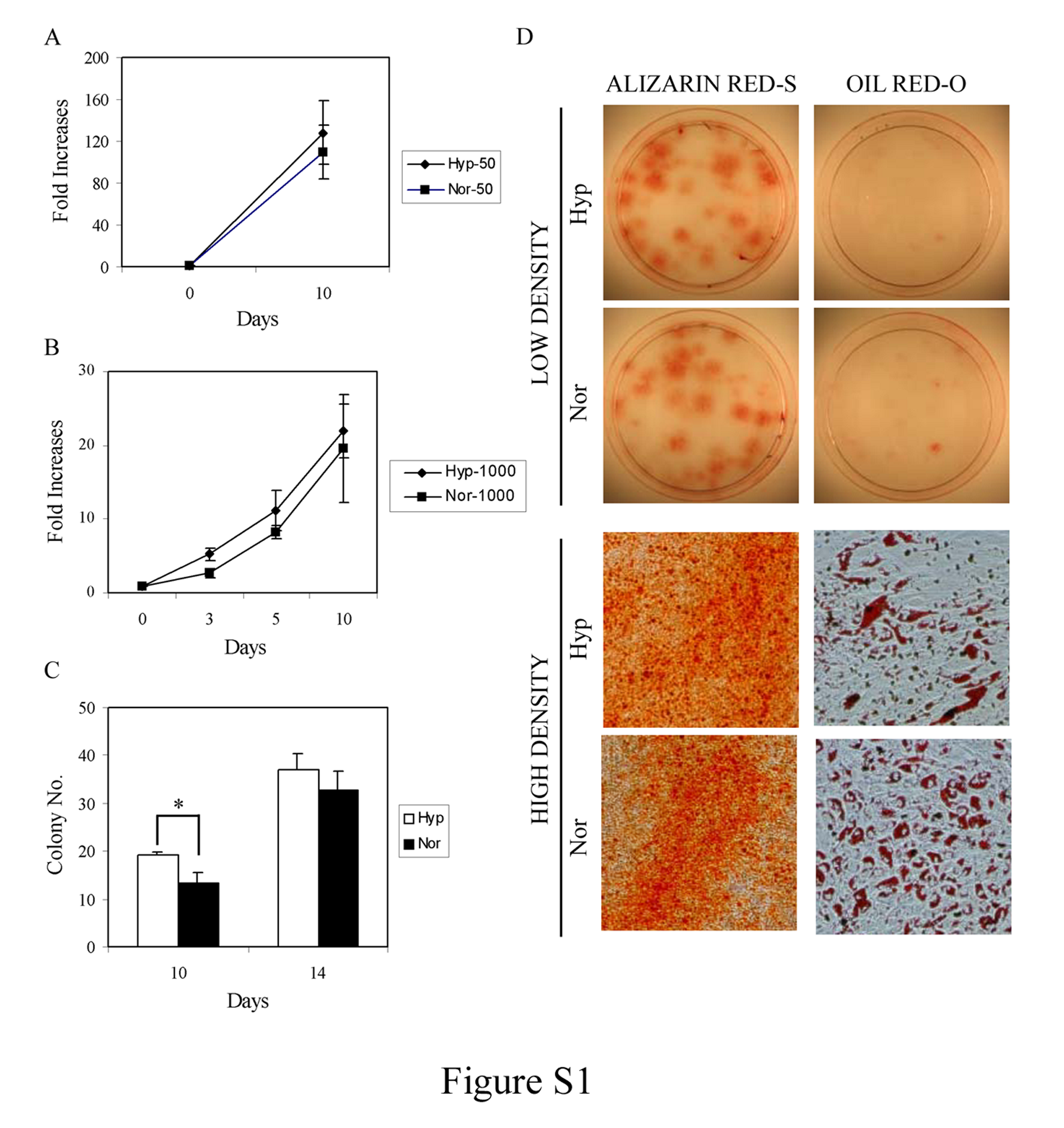

Supplement: Figure S1 — Reoxygenation reverses hypoxic effects on cell proliferation and differentiation. Passage 3 MSCs were plated at 50 cells/cm2, cultured in normoxic (Nor) or hypoxic (Hyp) conditions for 8 days, replated at (A) 50, (B) 1,000, (D-High Density panels) 10,000 cells/cm2 and (C, D-Low Density panels) 100 cells/60-cm2 dish and then cultured under normoxic conditions. (A, B) The cells were harvested and counted at 10 days (A) or at 3, 5 and 10 days (B). Graphs represent the fold increase in cell number per 60-cm2 dish. (C) Number of Crystal Violet-positive colonies formed at 10 and 14 days. Data are expressed as mean±standard deviation (n = 3). (*, p<0.05, Student's t test). (D-High Density panels) Cells were replated with induction medium the next day, cultured for additional 21 days and stained with Alizarin Red-S or Oil Red-O. (D-Low Density panels) Cells were cultured in CCM for 7 days and replated with adipogenic or osteogenic induction medium for additional 21 days. Osteogenic cells/colonies stained with Alizarin Red-S and adipogenic cells/colonies stained with Oil Red-O. (9.99 MB TIF) [file pone.0000416.s001.tif]

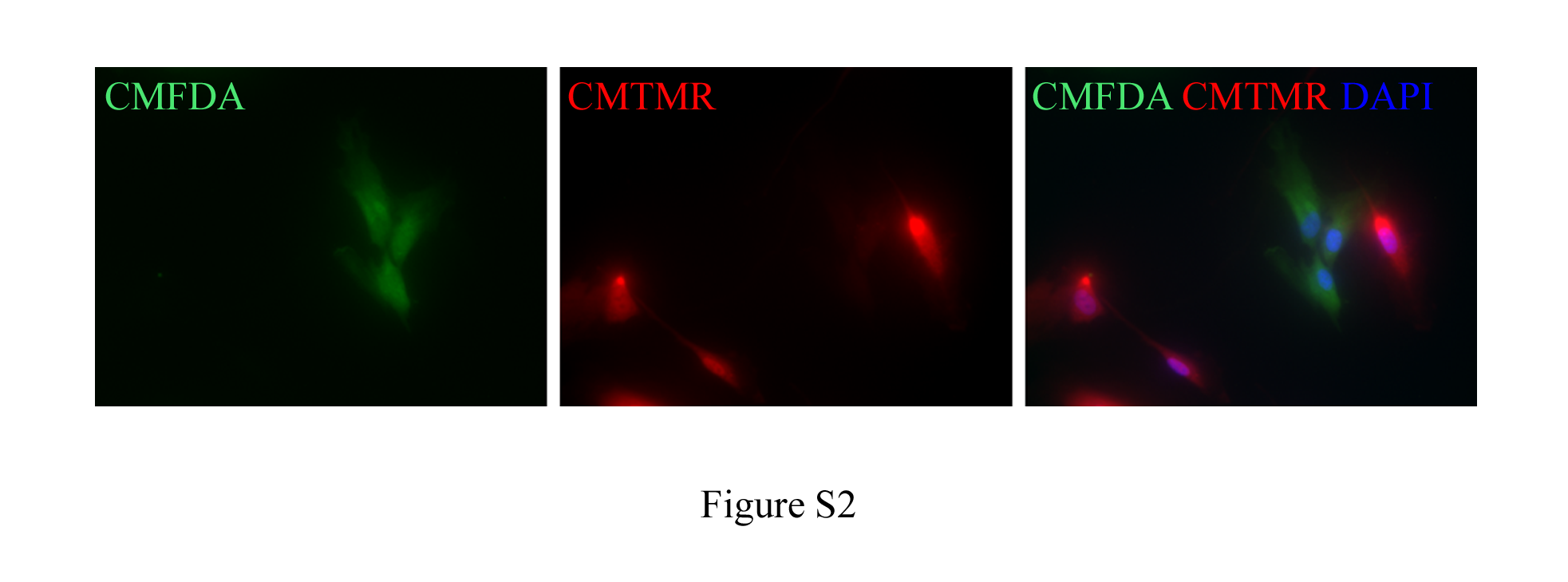

Supplement: Figure S2 — Detection of fluorescence after labeling with the vital dyes. Cells recovered from hypoxic and normoxic cultures were labeled with CMFDA and CMTMR, respectively. CMFDA-and CMTMR-labeled cells were then mixed at the ratio of 1 to 1 and incubated under a normal expansion condition. The cells were fixed and observed with an epifluorescence microscope 3 days later (200×magnification). (4.31 MB TIF) [file pone.0000416.s002.tif]
